# The Psychosocial Factors Affecting Chinese Outbound Exchange and Mobility Students’ Academic Performance During COVID-19

**DOI:** 10.3389/fpsyg.2022.872516

**Published:** 2022-08-09

**Authors:** Liu Li, Baijun Wu, Ataul Karim Patwary

**Affiliations:** ^1^School of Management, North Minzu University, Yinchuan, China; ^2^School of Marxism Studies, Chengde Medical University, Chengde, China; ^3^Faculty of Hospitality, Tourism and Wellness, Universiti Malaysia Kelantan, Kota Bharu, Malaysia

**Keywords:** COVID-19, psychosocial factors, personality, social support, language fluency, exchange and mobility students

## Abstract

COVID-19 has affected every aspect of our life, including economic, social, and academic. Exchange and mobility students face more difficulties overseas, and Chinese students are no exception. However, e-learning has been introduced by institutions in many countries. The present study examines the psychosocial factors affecting the academic performance of Chinese outbound exchange and mobility students during the COVID-19 pandemic. The study surveys about 186 Chinese outbound exchange and mobility students. The present study performs the quantitative data analysis using Partial Least Square Structural Equation Modeling (PLS-SEM) through the Smart PLS software version 3. By confirming the measurement model and structural model assessments, the study finds that personality, social support, and language fluency are psychosocial factors that significantly influence the exchange and mobility students’ academic performance. This study contributes by establishing relationships among psychosocial factors, language fluency and academic performance. Besides, practitioners can be benefitted by understanding students’ psychosocial factors and its relation to academic performance during COVID-19 pandemic.

## Introduction

The worldwide population is slowing down due to the spread of the current COVID-19 pandemic. Outbreaks of the new virus have spread to many nations worldwide (Gilbert et al., 2020). On March 11, 2020, the World Health Organization (WHO) publicly named the illness the 20th of March virus as “COVID-19.” Thousands of students have been affected by the COVID-19 pandemic spread across the globe, and the lockdowns of their schools have further shocked their families, and teachers. Thus, restricting students’ access to learning, especially in the absence of computers and internet facilities in many homes.

The COVID-19 pandemic is a public health emergency that has affected the education and other sectors of the economy, which made policymakers to take two main decisions. First, to close down all learning and education centers and environments to reduce close contact with COVID-19 positive patients and second, allowing workers in the manufacturing sector to continue operations in order to keep the economy active productively (Cairney, 2021). Many schools and colleges were forced to close due to government efforts to contain the spread of the COVID-19 as there were no immediate solutions to stop the spread of COVID-19 at its early phase. Student’s ability to learn has been harmed due to the closure of several universities across countries (Onyema et al., 2020). For international higher education, college students declined drastically worldwide (Yıldırım et al., 2021). The resultant effect is that learning centers e.g., colleges and universities adopt online learning platforms as alternative learning delivery mechanisms. This is to ensure the continuity of institutions and universities’ activities. Universities rely on learning management systems and open-source digital learning solutions to offer online courses. The old chalk-talk model has been replaced by new technology.

However, in online learning, university students have been facing difficulties in understanding, delivering, and managing online lessons, class assessments and laboratory experiments (Lassoued et al., 2020; Maatuk et al., 2021). There is a lot of trial and error and uncertainty for everyone regarding student assessments moving online (Burgess and Sievertsen, 2020). Several tests have been canceled outrightly. There are long-term consequences for the affected cohorts and an increase in inequality due to these interruptions. The students would feel alone, depressed, indifferent, or suicidal (Adewuya and Oladipo, 2020), posing a psychological anxiety in their online learning process. Psychosocial anxiety means an uncomfortable and stressful feeling that a person experiences during a stressed or stressful situation (Olimov and Maxmudova, 2022).

Many college students choose to study abroad. International higher education is expected to decline due to the closure of institutions and universities worldwide (Yıldırım et al., 2021).

Related life experiences can affect multiple cultures, but the perception of these events can affect individuals’ levels of suffering (Harkness and Monroe, 2016). There are 1.4 billion undergraduate students worldwide pursuing higher education (Bozkurt et al., 2020). To curb the spread of COVID-19, education, which is an important determinant of quality of life and a key to development, was particularly affected. After the WHO declared the outbreak a pandemic in March 2020 (Jebril, 2020), most countries worldwide closed all educational institutions to stop the pandemic from spreading further (Burgess and Sievertsen, 2020; Koh, 2020; Yulia, 2020). A report of UNESCO in 2020 estimates that school and learning space closures affect 94% of the world’s student population, with the percentage rising to 99 percent in low- and lower-middle-income countries (Diamond et al., 2020).

According to The Organisation for Economic Co-operation and Development [OECD] (2021), higher education has been severely impacted by COVID-19 pandemic as many universities had been closed and countries also closed the boarders. Even though, the higher education has been trying to replace face to face learning to online learning, there has been many challenges faced by students who does not have adequate accessibility of internet and gadgets (Tadesse and Muluye, 2020). Beside the learning facilities, in this pandemic, students also encounter the issues likewise personality (Rammstedt et al., 2022), social support (Saltzman et al., 2020) which might impact on their daily life and particularly on academic performance. Other than psychosocial factors, while teaching through online during this pandemic, language fluency has been an issue for the students that can influence on the academic performance (Diamond et al., 2020). Therefore, stress is triggered by changes in student’s life, coping capacity, and potential life risk (Heinen et al., 2017). Meanwhile, students are often dealing with this due to the difficulty of living in a new environment and a clear desire to excel in life which causes more anxiety problems among the students (Griep et al., 2016). Therefore, this study investigates the influence of Chinese mobility and exchange students’ psychosocial factors on their academic performances during the COVID-19 pandemic.

This paper aims to contribute by adding the new inputs in body of knowledge. Besides, it also can help the practitioners to get an insight on students’ psychosocial factors and its relation to academic performance. The remaining paper follows with literature review and hypotheses formulation, research framework, research method, results of the measurement and structural model, and discussion followed by implications of the study and conclusion.

## Literature Review and Hypothesis Development

### Theoretical Underpinning – Student Involvement Theory

In current study we mainly examined the role of psychosocial factors on academic performance of the students during COVID-19 pandemic. Since the basis of this study comprised with students’ involvement in academic activities, the research framework of the study underpinned by Student Involvement Theory (Austin et al., 1984). This theory initially developed by Austin et al. (1984) and modified in Austin (1999). Student Involvement Theory mainly emphasized on students’ involvement and development process in higher education. Previous studies also utilized this theory while explaining academic performance of students, personality, social support, and language fluency (Francis and Babu, 2019; Rivas et al., 2021). Therefore, this theory considers most suitable while examining the role of psychosocial factors on academic performance of the students during COVID-19 pandemic.

## Psychosocial Factors

### Personality

Baumert et al. (2019, pp.06) explained personality as “an individual’s characteristic patterns of thought, emotion, and behavior, together with the psychological mechanisms, hidden or not, behind those patterns.” Personality traits are a person’s ability to respond to a wide range of stimuli with the same level of thoughtfulness and determination, regardless of the context in which they arise (Thielmann et al., 2020). Personality also can be described in terms of these two dimensions, but two additional dimensions shed light on the concept of personality separate from those two (Tellegen and Waller, 2008; Matz and Harari, 2020). Individuals respond to vulnerabilities viewed as traits which triggered their personal behavior (Griva et al., 2013). Several studies have shown that research has been done on international students’ personality characteristics and psychosocial adjustment associations (Brunsting et al., 2018; Bulgan and Çiftçi, 2018; Xie et al., 2021). An individual’s ability to cope with stress and manage their emotions is strongly influenced by their internal sense of control (Galvin et al., 2018; Zhu et al., 2020). International students may affect the way they adjust psychologically due to the different personality traits of international students. Some multicultural or cross-cultural student affairs experts should consider that most of the time, due to stability, it’s too difficult to change it (Topcu et al., 2020). Research on personality is often done on an individual or small group basis without the primary framework in adjustment (Hogan and Sherman, 2020).

Schermer et al. (2020) stated that only a few studies are associated with personality factors in adjustment research. International students have different adjustment experiences due to differences in personality management of change. Bulgan and Çiftçi (2018) conducted a study in the United States with 243 participants to determine whether personality traits such as extraversion, agreeableness, conscientiousness, and openness are linked to better psychological and sociocultural adjustment. The study also found negative associations between neuroticism and better psychological and sociocultural adjustment and significant positive associations between these two variables (Hirai et al., 2015). Extensive studies on students and adults have found positive associations between extraversion (the ability to be pleasant to others), conscientiousness (the ability to pay attention to details), openness (the ability to be accepting of others), and their ability to adjust to their social environments.

### Social Support

Social support is considered an important element that helps international students develop a basic sense of adaptation to the new social climate (Jolly et al., 2021). Toker (2021) suggest that the decisive factor that plays a critical role in the current establishment abroad is social reinforcement, allowing the dilemma of new growth and transition problems to be discussed. In comparison, social reinforcement plays a crucial role in stress control. It helps control the framework and significant influences affecting interpersonal transition if somebody experiences ethnic or cross-cultural challenges (Ma et al., 2020).

Japanese scholars’ participation in extra-curricular activities will serve as a buffering for a cultural shock for American students. Student participation also matters study showing that the number of students who are fully engaged in the other tasks and seen more easily adjusted in new culture and students with low segment experience the same adjustment problem. They think that their anticipation and curiosity are distinguished from others to some degree. Ye et al. (2020) examines those other activities usually minimize mental tension and help obtain environmental and social support. There were seventy-four international scholars from Korea in the Pittsburgh area who concluded that the deep engagement is the explanation for obtaining high social support and because of the other behaviors that reduce their stress level, as that student. A significant contribution of American culture, language, and other personal relationships to obtain social support is the study’s finding. For instance, the psychosocial transition is easier and more feasible as they obtain social support due to other external events and interesting resources for international scholars. A study from United States of America concluded that international students from the same country will benefit from social help and some local students instruct them how to adapt to new ones (Bhochhibhoya et al., 2017). The institution must also provide international scholars within the campus with the organizational culture and give them opportunities for partition to obtain social support that aids in adaptation in that situation. Besides, universities should plan the style event that allows international students to engage with the host country’s community, such as developing structured and non-format activities, student orientation, and other services that enhance their social support level. Alsop and Bencze (2020) focused on the markers of melancholy and nervousness in students worldwide and observed that social assistance had a vital commitment to the paradigm in expecting wretchedness. Specifically, more elevated levels of depression were identified by students with lower levels of social support. In addition to rejection, they also discovered that social support tended to reduce nervousness, especially for students with lower social support levels who were more likely to have greater levels of discomfort. The imperative of using the social support structure to alleviate the detrimental impacts of cultural assimilation on mental prosperity has been demonstrated by late work (Shih et al., 2019).

Foreign students were helped socially with psychological well-being which plays a major role in controlling their community to build a positive atmosphere for social help (Baba and Hosoda, 2014). Due to COVID-19 pandemic, a significant number of students are facing a lack of happiness (Son et al., 2021). The efficiency of some of the better forms of social care that implicitly support international scholars as they suffer from psychological stress in prosperity (Kohls et al., 2021). Several studies suggest that international academic tension should be minimized with academic and social assistance (Maler et al., 2021). It may be possible to establish a network to provide cultural event facilitation and reduce stress relevant to the culture.

## Language Fluency

People who have a stronger connection with foreign nationals speak a more understandable language than those who do not (Guma and Jones, 2019). Language capacities are crucial for the social transition. Based on this premise, insightful foundations should offer and assist students in developing their English language fluency. According to scholars Oweis (2018), English capacity and change are related. Researchers also examined how language capability in English affects student success. In Mumford and Dikilitaş (2020), a group of researchers from Turkey deliberated the affecting factor from social introspection. Progress in these areas depended on proficiency with the English language. As the students’ English was higher than other students, they were happier in Britain. It is correct that most international students were joining from various countries; most are coming from Malaysia and have language as an obstacle. And a similar situation was faced by international students in Malaysia at the University of Malaya. The study needs a lot of time to understand the instructions provided by the teacher thoroughly. Around the same time, there are language assessment standards for international students from several different countries, including TOEFL (Test of English as a Foreign Language), IELTS (Academic Test of English), and The High-Intermediate Certificate of Cambridge English.

Most people define barriers from international scholars in terms of academic changes, language, and contact. The inability of international students to communicate with natives is a common issue, particularly in the test. Kruk (2018) believed that language anxiety had existed on the students’ simple change. According to Flesia et al. (2020), their low degree of stress is attributable to their well-educated in English and global communication skills. Formally, effective interactions play a critical role in academic adjustment to international. According to these researchers, participants’ assessment of fluency in English is affected by their English-speaking capacity. Competency in the language is of much significance for foreign academics.

## Academic Performance

**FIGURE 1:**
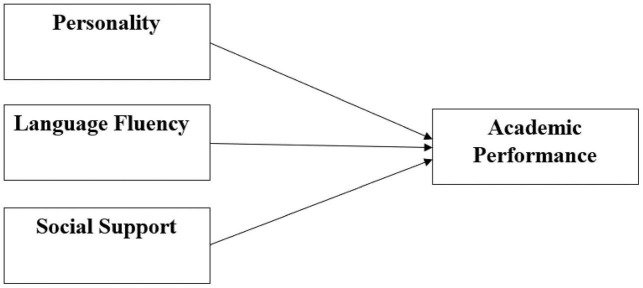
Research framework.

Social, neurological, and environmental conditions impact higher education (Chavoshi and Hamidi, 2019). According to previous work, students with the best academic records performed well in college (Okano et al., 2019). High school grades were great predictors of college grades (Bowen et al., 2018). More recent attempts have objectively evaluated student achievement, graduation, and teaching standards as distinct academic metrics of academic progress (Lee, 2018). As for student satisfaction, the effectiveness of instructional approaches and learning qualities can be ascertained (Almusharraf and Khahro, 2020). Student satisfaction is the positive assessment scores of learners about the diverse consequences and expectations associated with schooling. Study happiness is not consistent with academic achievement. A strong association was observed between students’ happiness and academic achievement; happy students are also significantly more successful (Lumontod, 2019). In contrast, students’ satisfaction is influenced by “non-cognitive” variables such as motivation and classroom communication rather than “cognitive” variables such as grades. At the same time, there is evidence that psychosocial traits. In contrast, personality and emotional intelligence can positively affect academic achievement and student progress, and the results are also inconsistent. Based on the above literature, this study has come out with the hypotheses and research framework (Please see Figure 1) below:

H1: *Personality has a positive influence on the academic performance of Chinese outbound exchange and mobility students.*

H2: *Language fluency positively influences Chinese outbound exchange and mobility students’ academic performance.*

H3: *Social support positively influences Chinese outbound exchange and mobility students’ academic performance.*

## Research Methods

The study adopts the quantitative approach to achieve the objectives of the paper. Besides, the study uses a questionnaire, designed on a 7-point Likert scale to elicit data on the variables contained in the research framework. A total of 200 questionnaires of the mobility psychosocial factors- academic performance scale was administered to Chinese outbound exchange and mobility students. A total of 186 questionnaires were found usable for the study after accounting for sample bias using straight lining for responses having standard deviations between 0.000 and 0.500. Table 1 below shows the references of the measurement items used for the constructs in this study.

**TABLE 1:** Questionnaire items.

Variables		Items
Personality Eysenck (2020) and Tellegen and Waller (2008)	1	I find it very easy to use train timetables, even if this involves several connections
	2	I find myself categorizing people into types (in my own mind).
	3	I like to collect a lot of different examples about something that I like, so I can see how they differ from each other
	4	I like to know the functions of committee members and how committees are structured.
	5	If I have a collection of circular date stamp, coins, other stamps, it would be highly organized.
	6	I am curious about the precise way buildings are constructed.
	7	I give directions for someone seeking to ask the way to any part of my hometown.
	8	I enjoy looking through catalogs of products to see the details of each product in terms of similarities and differences.
Language Fluency Birman and Trickett (2001), Takeuchi et al. (2002), and Spada and Lightbown (2009)		I like to know exactly which grammar point I am studying
		I believe my grammar will improve quickly if I communicate using English
		I find it easier to learn grammar when the instructor teaches it by himself or herself.
		I prefer lessons that focus on communication and grammar
		I like learning grammar through its explanations and practice exercises
Social Support Scott et al. (2011) and Gregory et al. (2010)	1	I have a special person around me whenever in need.
	2	I share my joys and sorrows with a special person.
	3	My family really tries to help me
	4	I get emotional help and support from my family
	5	I have a special person who is a real source of comfort to me
	6	My friends really try to help me
	7	I have friends with whom I can share my joys and sorrows I have friends with whom I share my joys and sorrows with.
	8	I can count on my friends when things go wrong
Academic Performance Mercugliano et al. (1999) and Yousef (2017)	1	I enjoy my academic lesson
	2	The way the lecturer speaks is important in understanding the lecture
	3	Lectures help me to identify my strengths and weaknesses
	4	The faculty has provided me with specific advice on how to improve my academic performance
	5	Lectures promote active reflection on the effectiveness of teaching
	6	The lectures encourage feedback that enhance learning
	7	Exam without marks prevents motivation for students cheating
	8	The time of the lectures are appropriate
	9	The pace (speed) of delivery of the teaching is reasonable.

## Measuring Instrument

The measuring instrument for the current study has two sections. Section A describes the demographic information of respondents including gender, marital status, age, current semester, and programme of study. Section B consists of the items on the main variables in the formulated hypotheses of the study (personality, language fluency, social support, and academic performance). Chinese exchange students rated their opinion for personality, language fluency, social support, and academic performance during COVID-19 pandemic on a seven-point Likert scale (“Strongly agree = 7” to “strongly disagree = 1”).

Personality is measured using eight (8) items adopted from Eysenck (2020) and modified by Tellegen and Waller (2008). The items include “I find it very easy to use train timetables, even if this involves several connections.” Five (5) items were adopted from Birman and Trickett (2001) and Takeuchi et al. (2002) to measure language fluency of the students. These items were previously used and validated by Spada and Lightbown (2009). The items include “I like to know exactly which grammar point I am studying.” Social support was measured by eight (8) items adopted from Scott et al. (2011) and Gregory et al. (2010). The items include “There is a special person who is around when I am in need.” Academic performance was measured by nine (9) items adopted from Mercugliano et al. (1999). These items were previously modified and validated by Yousef (2017). The items include “The way the lecturer speaks is important in understanding the lecture.”

## Results and Findings

### Respondents’ Information

Table 2 represents the respondents’ demographic distribution for gender, marital status, age, and the number of semesters completed by students’ exchange and mobility. Out of 186 respondents, 62.4% (116) respondents are male, and 37.6% (70) are female. The majority of the respondents are single, 84.9% (158), and married are 14% (26). In terms of age group, the highest age group consists of “20–22 years old” 44.1% (82) followed by “17–19 years old” which is 23.1% (43), “23–25 years old” 15.6% (29) “29 years old and above” is 10.2% (19), and 26–28 years old 7.2% (13). Students are currently in different semesters of their study; many of them are from semester 1 to 2, which is 44.6% (83), followed by semester 5 to 6 is 32.8% (61), semester 7 to 8 is 17.7% (33) and remaining is semester 3 to 4, 4.8% (9). For the program of the study, majority of them are undergraduate students (72.6), followed by masters 20.4% and Ph.D. is 7%.

**TABLE 2:** Demographic profile of the respondents.

Respondents’ information	Frequency	Percentage
**Gender**		
Male	116	62.4
Female	70	37.6
Total	186	100
**Marital status**		
Single	158	84.9
Married	26	14.0
Divorced/widowed	2	1.1
Total	186	100
**Age (years)**		
17–19 years old	43	23.1
20–22 years old	82	44.1
23–25 years old	29	15.6
26–28 years old	13	7.0
29 years old and above	19	10.2
Total	186	100
**Semester**		
Semester 1–2	83	44.6
Semester 3–4	9	4.8
Semester 5–6	61	32.8
Semester 7–8	33	17.7
**Programme of the study**		
Undergraduate	135	72.6
Masters	38	20.4
PhD	13	7
Total	186	100

### Measurement Model Assessment

**FIGURE 2:**
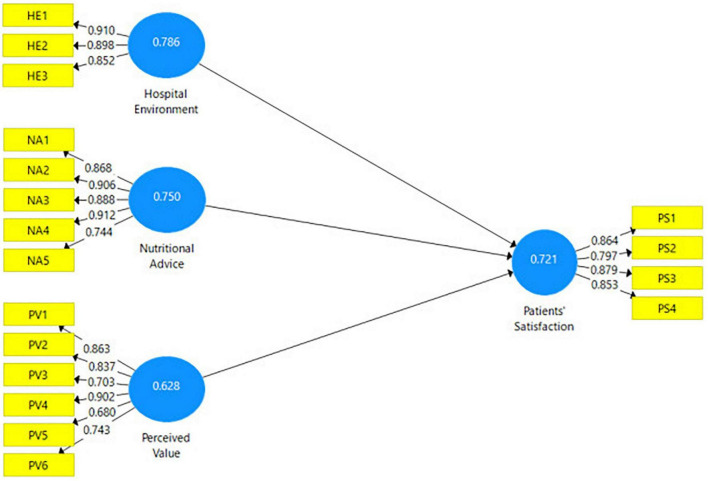
Measurement model.

For the data analysis, the researcher used Smart-PLS version 3.0. Following Dijkstra and Henseler (2015), a consistent algorithm was applied to evaluate the convergent and divergent validity of the variables used in the study. All the items should load at least 0.60, composite reliability (CR) more than 0.70, and average variance extracted (AVE) above 0.50 to confirm convergent validity (Dijkstra and Henseler, 2015). As shown in Table 3 and Figure 2, the four variables used in this study are reliability and validity. The reliability measure (Table 3) where any single variable meets the conditions is Cronbach alpha. It implies that all variables included in the analysis have passed the validity reliability arguments. Besides, CR was above the recommended value, while the extracted average variance was above 0.50.

**TABLE 3:** Construct validity and reliability.

Variables	Cronbach’s alpha	Composite reliability	Average variance extracted (AVE)
Academic performance	0.930	0.942	0.643
Language fluency	0.836	0.890	0.627
Personality	0.933	0.943	0.675
Social support	0.886	0.909	0.564

In Table 4, the Heterotrait-Monotrait Ratio is considered further to assess discriminant validity; the Smart-PLS assessed the latest method. HTML ratio is shown in Table 4 to confirm that all the variable meets certain conditions as their correlations do not exceed the value of 0.85.

**TABLE 4:** Heterotrait-Monotrait Ratio (HTMT).

Variables	1	2	3	4
1. Academic performance	–			
2. Language fluency	0.338			
3. Personality	0.196	0.156		
4. Social support	0.157	0.190	0.086	–

In Table 5, variance inflation factors (VIFs) are used to assess any high collinearity among the constructs’ items. Based on the outer loadings, the measurement model found only “Personality 1” to have more collinearity with one of the study items, which is acceptable since no other items showed high collinearity.

**TABLE 5:** Variance inflation factor (VIF).

Construct	Items	VIF
Academic performance	Academic 1	3.320
	Academic 2	2.829
	Academic 3	2.242
	Academic 4	2.753
	Academic 5	2.607
	Academic 6	2.245
	Academic 7	3.088
	Academic 8	2.620
	Academic 9	1.950
Language fluency	Language 1	1.100
	Language 2	2.440
	Language 3	3.241
	Language 4	3.407
	Language 5	3.490
Personality	Personality 1	5.112
	Personality 2	4.634
	Personality 3	3.268
	Personality 4	4.116
	Personality 5	4.252
	Personality 6	2.322
	Personality 7	3.073
	Personality 8	4.595
Social support	S. Support 1	2.806
	S. Support 2	2.916
	S. Support 3	2.617
	S. Support 4	2.449
	S. Support 5	2.526
	S. Support 6	1.893
	S. Support 7	1.976
	S. Support 8	1.208

### Structural Model Assessment

**FIGURE 3:**
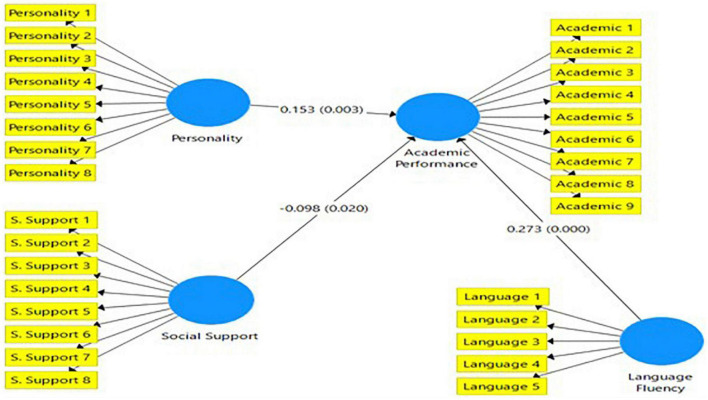
Structural model.

We have used statistical method (i.e., *p*- values, *t*-values) and bootstrapping suggested by Zhao et al. (2010), Nitzl et al. (2016), and Rasoolimanesh et al. (2021) to test indirect effects. The study uses the bootstrapping method to assess the structural model to determine the direct effects of language fluency, personality, and social support on the academic performance of Chinese outbound exchange and mobility students during the COVID-19 pandemic (March 2021–July 2021). Table 6 and Figure 3 shown that language fluency (Beta = 0.273; *p*-value = 0.000 < 1%; *t*-value = 5.517), personality (Beta = 0.153; *p*-value = 0.004 < 1%; *t*-value = 2.907), and social support (Beta = −0.098; *p*-value = 0.016 < 5%; *t*-value = 2.414) have significant influences on students’ academic performance. However, the relationship between social support and students’ academic performance is negatively significant.

**TABLE 6:** Direct effects of the antecedent variables of students’ academic performance.

Relationships	Path coefficient (Beta)	Standard deviation	*t*-value	*P*-value
Language fluency → academic performance	0.273	0.050	5.517	0.000
Personality → academic performance	0.153	0.053	2.907	0.004
Social support → academic performance	–0.098	0.041	2.414	0.016

### Goodness of Fit

Concerning the model fitness of the constructs used in this study, this study considers SRMR values as the most important criteria. Although, Hair et al. (2016) concluded that it is unnecessary to examine model fitness in Smart PLS using SRMR values, Table 7 implies that SRMR values meet model fitness criteria regarding this study.

**TABLE 7:** Model fitness summary.

	Saturated model	Estimated model
SRMR	0.059	0.059
d_ULS	1.602	1.602
d_G	0.874	0.874
Chi-square	1625.213	1625.213
NFI	0.792	0.792

## Discussion

Due to the global pandemic since March 2020, students worldwide were feeling anxiety, fear of academic failure, and fear of academic year loss, which can have a significant impact on the overall academic performances of the students. Consequently, the COVID-19 pandemic has brought considerable changes in the overall academic environment, making the students uncomfortable to cope with the new academic environment and badly impacting the students’ academic performance (Vargas et al., 2020). Nevertheless, the impact of the COVID-19 pandemic becomes more acute for the mobility and outbound exchange Chinese students as they come only for a short period to complete either one or two academic semesters in other abroad universities. Hence, this study examines how COVID-19 impacts the academic performance of the Chinese outbound exchange and mobility students. Further, this study examines the relationship between personality, language fluency, social support, and academic performance of Chinese outbound exchange and mobility students during the COVID-19 pandemic. To achieve this objective, the researcher has three main constructive hypotheses in this study. First and foremost, this study considers the students’ personality as one of the main factors that, either positively or negatively, can impact the students’ academic performances. Hence, the very first hypothesis of this study is that personality has a significant influence on the academic performance of Chinese outbound exchange and mobility students.

Furthermore, language fluency is another crucial variable that has been given attention in this study, assuming that language fluency can significantly impact students’ academic performance. Hence, the second hypothesis of this study articulates that language fluency has a significant influence on Chinese outbound exchange and mobility students’ academic performance. Furthermore, besides the personalities and language fluency of the Chinese outbound exchange and mobility students’, social support is one of the main factors that highly influence overall academic performance. Therefore, the third hypothesis of this study is that social support has a significant influence on Chinese outbound exchange and mobility students’ academic performance during the COVID-19 period. Moreover, to examine the basement of the above mentioned three hypotheses, this study utilized the PLS-SEM. Based on the analysis through structural equation modeling and PLS-SEM, all the three main hypotheses of this study were accepted based on the threshold provided by Hair et al. (2017) and Patwary et al. (2021).

As far as the first hypothesis is concerned, the thorough analysis of this study shows that students’ personality has a significant favorable influence on the academic performance of the Chinese mobility students studying in abroad countries. The findings of this study imply that students who possess strong personalities can deal with emotional, psychological and physical problems efficiently during the pandemic time, which help them to perform consistently better in their academic examinations. Further, several previous studies also show that students’ personalities positively influence the students’ academic performance (Yousef, 2017; Andersen et al., 2020; Fuertes et al., 2020). Further, the second hypothesis of this study confirms that those students who have higher language fluency tend to achieve more academic success regardless of the pandemic. The finding of this study assures that students who have reasonable control over their language expertise can understand better the given task during the classes, which help them to do the assignment and another class task properly. Moreover, students with English language fluency can communicate better with their teachers and classmates, which allow them to perform consistently better in the class tasks and during the exams. Previous studies conducted by Oducado et al. (2020) and Thomas (2020) found similar results that language fluency increases students’ level of confidence which in return helps the students to achieve better academic performance.

Lastly, the third hypothesis results posit that the social support around students negatively affects the students’ academic performances. During COVID-19, the Chinese outbound exchange and mobility students have been suffering abroad due to the lack of proper social support. Since, to tackle the pandemic situation, most countries worldwide have implemented lockdown regulations that have prevented any types of physical, social support. Hence, students are also prohibited from any social gathering, making the students feel more lonely, isolated, and helpless. Therefore, due to the lack of social support, students are emotionally weak and perform poorly in their academic results. Further, the findings of the recent studies also supported the third hypothesis of this study, which states that lack of social support around the students negatively affects the students’ academic performance (Camacho et al., 2021; Putra et al., 2021).

Thus, the findings of this study show that students’ personality and language fluency positively influence the academic performances of the Chinese outbound exchange and mobility students. On the other hand, lack of social support negatively impacts the overall academic performances of the Chinese outbound mobility students during the period of COVID-19.

## Theoretical Implications

First and foremost, the core contribution of this empirical study focuses on the theoretical perspective in the research area of psychological factors affecting the performance of mobility students. In previous studies, most researchers have examined anxiety, stress, depression, and emotion as psychological factors that affect students’ academic performances. For instance, Hasan and Bao (2020) have utilized the psychological distress model, which can impact students’ academic performance. Further, Vargas et al. (2020) have utilized noise, temperature level, and lighting as independent variables that can affect the academic performance of university students. In addition, Eya et al. (2020) have employed attitude, motivation, and self-regulation as socio-psychological factors that can impact the students’ academic performance.

Further, none of the previous empirical studies has implemented the above mentioned three psychological factors to explore the academic performance of the mobility students.

However, in the context of this study, the author has introduced new variables as psychological factors that can directly or indirectly impact the academic performance of mobility and outbound exchange Chinese students. Moreover, the three main psychological factors introduced in this empirical study are personality, language fluency and social support. Furthermore, this study has validated the eight items introduced by Eysenck (2020) for the variable personality. Moreover, this study also verified eight items for the variable social support developed by Schieman (2005). Also, it validated five items for the factor language fluency, which is taken from Birman and Trickett (2001). Hence, this study has added a new horizon in the perspective of psychological factors affecting the academic performance of mobility and outbound exchange Chinese students.

## Practical and Policy Implications

Looking at the practical implication of this study, this is one of the very first initiatives in the empirical study addressing the very recent COVID-19 pandemic phenomenon by introducing psychological factors such as personality, language fluency and social support affecting the academic performances of the Chinese mobility and outbound students. Usually, mobility students go abroad for a very short period to study one or two semesters in another university to experience different cultures and environments. Moreover, it is always challenging for mobility students to maintain excellent academic performance in a new environment (Aresi et al., 2021). Nevertheless, the recent COVID-19 pandemic has made it further difficult for mobility students to achieve better academic performance. Therefore, this study would examine how psychological factors such as social support and personality affect the academic performance of the Chinese mobility and outbound students during COVID-19. Besides psychosocial factors, language fluency is also a crucial factor for students that has significant influence on their academic performance (Diamond et al., 2020). Therefore, this study also examines the role of language fluency on their academic performance during COVID-19 pandemic.

Further, concerning the policy implication of this study, the findings of this study would assist the administrators and executives responsible for maintaining the overall well-being of the Chinese mobility and outbound students studying abroad. Moreover, the responsible administrators taking care of Chinese mobility and outbound students can use the findings of this empirical study to develop a new policy that can help the Chinese mobility students enhance their academic performances. Furthermore, the welcoming institutes and universities which invite the Chinese mobility students can also utilize the findings of this empirical study to identify the psychological factors which affect the academic performances of the mobility students. Thus, the valuable results of this study can help the administrators and institutions develop the rules and policies that can contribute toward achieving better academic performances for the Chinese mobility students.

## Limitation and Future Study Directions

This empirical study has many significant contributions in the context of psychological factors impacting the academic performances of the Chinese exchange and mobility students. However, this study has also a few limitations to be addressed in further studies. Firstly, due to the current pandemic situation, the data for this study was collected from purposively targeted two hundred sample students. Thus, future studies can address an enormous population to achieve more diverse and accurate results. Secondly, this study only focuses on the Chinese exchange students, which are a very narrow part of the group. Hence, the findings of this study might only address the problems faced by the Chinese exchange students. Therefore, the future study can also include other countries exchange students to achieve the findings, which will address the overall exchange and mobility students’ psychological factors affecting their academic performances. Lastly, another limitation of this study is that this study applied a simplified model for conceptualizing the results. Consequently, the future study can include more psychological factors as independent variables. Also, it can include the moderating or mediating variables in the model to investigate the existed models from different aspects.

## Conclusion

To sum up, this empirical study inspects the impact of COVID-19 on the academic performance of the Chinese mobility and exchange students from three main perspectives, namely, personality, language, and social support. The findings of this study assure that Chinese exchange students’ personality and language fluency has a significant positive relationship with academic performance. However, the lack of social support around the Chinese exchange students due to the COVID-19 situation has a negative relationship with academic performance. Hence, the findings of this study exhibit that psychological factors, personality, language fluency and social support have significant impacts on the overall academic performances of the Chinese mobility and exchange students. This study expects that the findings of this empirical study would help the Chinese exchange student’s administrators and institutions have a better understanding of the psychological factors affecting students’ academic performances. Therefore, based on the findings of this study, the institution can take the necessary steps to help the Chinese mobility and exchange students achieve better academic performances and maintain students’ overall wellbeing.

## Data Availability Statement

The raw data supporting the conclusions of this article will be made available by the authors, without undue reservation.

## Author Contributions

All authors listed have made a substantial, direct, and intellectual contribution to the work, and approved it for publication.

## Conflict of Interest

The authors declare that the research was conducted in the absence of any commercial or financial relationships that could be construed as a potential conflict of interest.

## Publisher’s Note

All claims expressed in this article are solely those of the authors and do not necessarily represent those of their affiliated organizations, or those of the publisher, the editors and the reviewers. Any product that may be evaluated in this article, or claim that may be made by its manufacturer, is not guaranteed or endorsed by the publisher.
